# Delay in seeking healthcare for pneumonia and associated factors among mothers/caregivers of children aged 2–59 months in public health facilities in Nekemte town, Ethiopia

**DOI:** 10.1186/s12887-022-03825-x

**Published:** 2023-01-12

**Authors:** Dereje Temsesgen, Berhanu Wordofa, Tewodros Tesfaye, Werku Etafa

**Affiliations:** 1 Department of Pediatrics and Child Health Nursing, Institute of Health Sciences, Wallaga University, Nekemte, Ethiopia; 2 Psychiatry Nursing, College of Health Science, Addis Ababa University, Addis Ababa, Ethiopia; 3 Pediatric Program and Child Health Nursing, College of Health Science, Addis Ababa University, Addis Ababa, Ethiopia

**Keywords:** Delay in seeking healthcare, Pneumonia, Children aged 2-59 months, Ethiopia

## Abstract

**Background:**

Pneumonia is the most significant infectious disease and the predominant cause of death among under-five children (U5C) in low- and middle-income countries. It is the second leading cause of death in Ethiopia. Delay in seeking healthcare is one of the contributing factors to pneumonia-associated mortality. There is a limitation to the study aimed at identifying health-seeking behavior and risk factors in the western part of Ethiopia. The study aimed to determine the level of delay in seeking healthcare for pneumonia and associated factors among caregivers of U5C in public health facilities in Nekemte town, Ethiopia.

**Methods:**

A health facility-based cross-sectional study was conducted from 1st March to 5^th^April, 2022 using a structured interviewer-administered questionnaire to collect data from 410 caregivers of children aged 2–59 months. We used a systematic sampling technique for collecting the data. For analysis, data were entered into Epi Data version 4.6 and exported to SPSS version 25. Binary logistic regression was used to identify the associated factors of delay in seeking healthcare for pneumonia at a *p*-value < 0.05 using a 95% confidence interval (CI) in multivariable logistic regression.

**Results:**

A proportion of delays in seeking healthcare for pneumonia among children aged 2-59 months is 62.2%. Rural residence (AOR = 2.77, CI:2.48-5.17), child aged ≥12 months (AOR = 5.4,95%CI:4.17-7.20), monthly income < 1000 Ethiopian birr (AOR = 6.11,95%CI:2.16-17.26,), not using health insurance (AOR = 8.93,95%CI:5.43-14.68), use of self-medication (AOR = 10.97,95%CI:1.85-65.3), poor knowledge (AOR = 4.63,95%CI: 1.35-15.9), perceiving illness due to pneumonia as mild (AOR = 14.97,95%CI:9.76-22.9) and no previous admission history (AOR = 2.85,95%CI:1.77-4.56) were significant factors for delay in seeking healthcare for pneumonia among children aged 2-59 months.

**Conclusion:**

The study emphasizes that caregivers’ delay in seeking healthcare for pneumonia is high. Creating caregivers’ awareness or providing adequate health education to develop early healthcare-seeking behavior and encouraging caregivers to use health insurance is essential.

## Introduction

In low- and middle-income nations, pneumonia is the primary infectious cause of mortality and morbidity [1]. In children aged less than 59 months, the burden of pneumonia remains high due to the younger age of the child associated with low immunity, pre-existing diseases, malnutrition, air pollution, missing vaccinations, and environmental hazards like (indoor air pollution, overcrowding, and smoking) [1–3]. Although several microorganisms are the etiologic agents of pneumonia, *Streptococcus pneumonia* and *H. influenzae* are the most common bacteria that cause pneumonia [2]. The respiratory syncytial virus is the most common type of virus that causes pneumonia in younger children [3].

Complications of pneumonia are categorized either as local (parapneumonic effusion, empyema, necrotizing pneumonia, and lung abscess) or as systemic (bacteremia, metastatic infection, multiorgan failure, acute respiratory distress syndrome, disseminated intravascular coagulation and, rarely, death) [4]. However, children from pneumonia can be protected (through effective use of exclusive breastfeeding and complementary feeding), prevented (by vaccination, hand washing with soap, reducing household air pollution, and preventing infectious diseases), and treated by the appropriate therapies, including supplemented oxygen [3].

Globally, almost 1 million children under the age of five die from pneumonia every year, accounting for 15% of all child deaths, while South Asia and sub-Saharan Africa are predominantly affected by this infectious disease [3]. Africa loses thousands of children due to pneumonia each year that causes around 750,000 child deaths per year in sub-Saharan African countries [3]. In Ethiopia, pneumonia kills 15 people per 1000 live births, and the country ranks sixth among the top 15 countries in the world for pneumonia morbidity and mortality [5].

Primary caregivers’ timely recognition of the signs and symptoms, as well as subsequent care seeking for treatment, are poor, resulting in many under-five deaths in developing countries despite effective treatment for pneumonia exists [6]. As a result, the World Health Organization (WHO) estimates that prompt medical attention can save 20% of children’s lives and dramatically reduce morbidity from acute respiratory infections, including pneumonia [7]. Delaying seeking healthcare for pneumonia contributes to many deaths in developing countries [8]. Around the world, 40% of kids put off getting the aid and care they need, but in sub-Saharan Africa, where pneumonia kills the most kids, that number rises to almost 60% [9]. In our country, Ethiopia, health-seeking behavior is deficient in around 61.3% of caregivers [10].

According to a WHO report, more than half of childhood mortality is caused by a delay or insufficient response to medical care, which can be avoided by obtaining medical help sooner [11]. Despite the fact that it is recommended that children seek immediate medical attention, approximately 40% of children worldwide do not receive the necessary aid and care, and approximately more than half of children in sub-Saharan Africa, where the majority of pneumonia deaths occur, are left at home or delay in seeking care [9].

According to a United Nations International Children Emergency Fund (UNICEF) report, just 3 out of every five children in Sub-Saharan Africa with pneumonia-specific symptoms are not early taken to an appropriate health facility for treatment; children from poorer and less educated families are less likely to seek care [12]. Accordingly, in Chad (27.4%), Central African Republic 30.9%, Democratic Republic of Congo (44.2%), Nigeria (41.9%), Sierra Leone (73.2%) and Malawi (68.8%) seek health care on time respectively whereas the remained delayed to seek care from the health facility for their children [9]. The Ethiopian Demographic Health Survey (EDHS) 2016 report showed that about 70% of mothers with less than 5 years old do not seek healthcare promptly without delay for pneumonia (acute respiratory infections) in Ethiopia [12].

According to earlier research [13, 14], mothers’ and caregivers’ health-seeking for pneumonia in children between the ages of 2-59 months is influenced by socio-demographic factors, such as parental socioeconomic status, health facility-related factors (such as distance from the health facility), and lack of awareness of pneumonia.

In agreement with the WHO, the Ethiopian government is implementing integrated community case management to reduce childhood-related mortality and morbidity due to pneumonia [15]. The Ethiopian government is working to improve the survival of children under five [16]. For example, training healthcare workers, formulating case management guidelines, strengthening communities with health insurance services and adequate health information, and extending healthcare facilities and infrastructures for implementing integrated community case management.

However, from our experience, caregivers’ practices like taking their baby to church, using traditional methods, and using types of syrup previously bought for unrelated cases are reported. A community uses different beliefs, cultures, and traditional ways of managing childhood illness. Some of them are toxic to child growth and development. The Ethiopian government’s commitment alone is not sufficient to reduce childhood mortality and morbidity due to pneumonia. There is limited research sought to identify the level and contributing factors related to delay in healthcare seeking for pneumonia in Ethiopia. Therefore, this study desired to assess the level of delay in healthcare seeking for pneumonia and associated factors among caregivers of U5C in public health facilities in Nekemte town, Ethiopia.

## Methods

### Study area and period

The study was conducted in public health facilities in Nekemte town from 1st March to 5th April, 2022. Nekemte is the capital town of East Wollega which is found about 331 km away from Addis Ababa, the capital city of Ethiopia. There are four public health facilities in the town: Nekemte Specialized Hospital, Wollega University Referral Hospital, Nekemte and Chalalaki health centers. The estimated number of under five children in Nekemte town is 34, 334.

### Study populations

All mothers/caregivers of children aged 2-59 months with pneumonia who visited public health facilities in Nekemte town were source populations, while mothers/caregivers of children aged 2-59 months with pneumonia during the study period were the study populations.

### Eligibility

Mothers and caregivers of children aged 2-59 months who had pneumonia signs and symptoms and had visited a healthcare facility to receive treatment were included, but those whose children were receiving follow-up care for pneumonia cases were excluded. In addition, mothers or caregivers who were not volunteer and unable to communicate for medical and other cases were also excluded.

### Sample size determination and sampling procedure

Using a single population proportion method, a 95% confidence interval, a 5% margin of error, and the proportion (p) of patients who delayed seeking treatment for pneumonia (48.6%) from a study conducted in Bahir Dar city, the sample size was computed [17]. So, after adjusting for the non-response rate (10%), the total sample size was adjusted to 422.

To obtain the requisite sample size, the study encompassed all public health institutions in Nekemte town. The necessary sample size from each healthcare facility was calculated using a proportional allocation. Accordingly, for the aim of the study, estimates were made from Nekemte Specialized Hospital (163), Wollega University Referral Hospital (155), Nekemte Health Center (54) and Chalalaki Health Center (50). To gather the data for the study, a systematic sampling technique was used. In order to select 422 study participants from all of the health facilities, a systematic sampling technique was used to select each study subject at every k^th^ interval (k = N/n, where N is the average monthly number of pneumonia cases among children aged 2-59 months in all health facilities, n is sample size, and k is an interval). To calculate the overall number of pneumonia cases in 1 month across all public health facilities, we utilized the average number of cases of pneumonia over the previous 3 months (753). Next, using the result of the formula k = 753/422 (1.782). Based on their daily visits, study participants in each facility were chosen every two intervals.

## Study variables

### Dependent variable

Delay in healthcare seeking for pneumonia in children aged 2-59 months.

### Independent variables

The independent variables included socio-demographic factors (caregivers’ age, gender, monthly income, place of residence, family size, occupation, religion, and marital status, as well as the age and gender of the children), facility-related factors (type of health facility, mode of transportation, and distance to the nearest health facility for mothers, cost for treatment, and waiting time), and caregivers’ knowledge (previous knowledge of seeking medical attention, warning signs, symptoms, and signs of pneumonia, among other things).

### Operational definitions

Delay in seeking healthcare for pneumonia: seeking healthcare at the public health facility after 24 hours from when the caregiver recognized the signs and symptoms of pneumonia, including cough and fast breathing [17–19].

Promptness in seeking healthcare: seeking healthcare for pneumonia at a public health facility within 24 hours from when the mother/caregiver recognized the signs and symptoms of pneumonia, including cough and fast breathing [17–19].

Healthcare: treatment provided by a recognized public health facility that offers services for promotion, prevention, and healing [20].

Self-medication: is when mothers identify their children’s illness, initiate all treatment options, and use them without a medical prescription [20].

Traditional medicine: Patients who appear to be ill or sick are treated using spiritual, religious, and practice-based knowledge [20].

Good knowledge: Mothers or caregivers who correctly answer ≥80% of knowledge assessing questions [17].

Poor knowledge: Mothers or caregivers who correctly answer < 80% of knowledge assessing questions [17].

Severe pneumonia: Severe respiratory distress (e.g., grunting, extremely severe chest pain), central cyanosis (oxygen saturation 90%), or indicators of pneumonia with a general danger sign (inability to breastfeed or drink, lethargy or lowered level of consciousness, convulsions) are all indications of severe pneumonia [21].

Non-severe pneumonia: Cough and rapid breathing (if it is less than 50 breaths per minute in children aged 2 to 11 months and < 40 breaths per minute in children aged 1 to 5 years) or cough and chest in a drawing without danger indications are symptoms of non-severe pneumonia [21].

### Data collection procedure and quality control

Data was gathered at U5C’s outpatient departments after doctors diagnosed the young patient with pneumonia (OPD) by speaking with the mothers or other caregivers in person. Four nurses with a first degree were given the task of gathering data. One supervisor enrolled in a master’s program in nursing was given the charge of overseeing data gathering. The supervisor and the data collectors received training before the data was collected.

A modified and customized version of a previous study was used. The tool was created in English, translated into the local language of Afaan Oromo, and then utilized for the interview. Data collectors and study participants would find the instrument simple to use. After that, the translated Afaan Oromo version was used to analyze the information gathered. The data collection instrument consists of a socio-demographic questionnaire (13 questions), a questionnaire about health facilities (9 questions), a questionnaire about knowledge (14 questions), and a questionnaire about sickness features (6 questions). Three answers (yes, no, and I don’t know) were provided for knowledge-testing questions. Mothers/caregivers who correctly responded to Mothers/caregivers who correctly answered the item were given one (1) and those answered incorrectly and I don’t know were given zero (0). Mothers or caregivers who answered the item correctly scored one point, while who could not answer correctly scored zero. This resulted in a final score between 0 and 14. Zero (0) and 14 scores represent mothers or caregivers who incorrectly and accurately answered all knowledge testing items, respectively. Finally, the score was converted to a percentage to dichotomize mothers’ or caregivers’ knowledge as good vs. poor).

The questionnaire was pretested on 5% (*n* = 21) of the sample size at Bako Hospital and Sire Health Center. Outside experts confirmed the validity of its contents. The tool was modified in response to feedback from subject-matter experts as well as the results of a pretest.

### Data processing and analysis

The data were entered into the statistical software Epi-Data version 4.6 and exported to SPSS version 25.0 for analysis. We used both descriptive and inferential statistics. An odds ratio with a 95% confidence interval was used to test the relationship between the independent and outcome variables. Variables with a *p*-value of < 0.25 in the bivariate analysis were considered for multivariate logistic regression. A multivariable logistic regression determined the significant variable at a p-value of < 0.05 was used.

## Results

### Socio-demographic characteristics of the respondents and their children

A total of 410 study participants completed the interview in this study, yielding a response rate of 97.16%.The respondents ranged in age from 15 to 48 years, and the most frequent age group was 25-34, which accounted for 240 (58.5%). The mean (SD) age of study participants was 28.71 ((±6.053) years, whereas the mean (SD) age of children was 23.73 (14.313) months. Females made up 404 (98.5%) of the study participants, while boys made up 178 (43.4%). About 315 (76.8%) of the study participants were rural residents, whereas the rest were from urban areas. Most of the study participants (95.1%) were married. The vast majority (78.5%) of them were protestants. Many mothers’ educational statuses are in secondary school (43.9%). Of the study participants, 108 (26.3%) were merchants, and the average monthly income of the study participants was 2947.68 ETB (Table 1).

**Table 1:** Socio-demographic characteristics of the study respondents and their children in public health facilities in Nekemte town, Ethiopia, 2022

Variable	Category	Frequency	Percentage (%)
Age of mother/caregiver in years	15-24	96	23.4
	25-34	240	58.5
	35-44	67	16.3
	> 45	7	1.7
Gender of caregiver	Male	6	1.5
	Female	404	98.5
Residence of caregiver	Urban	95	23.2
	Rural	315	76.8
Marital status of the caregiver	Married	390	95
	Unmarried	4	1.0
	Divorced	8	2.0
	Widowed	8	2.0
Religion of caregiver	Protestant	322	78.5
	Muslim	33	8.0
	Orthodox	39	9.5
	Others	16	4
Educational status of caregiver	Unable to read and write	19	4.6
	Primary education	91	22.2
	Secondary education	180	43.9
	College and above	120	29.3
Number of children aged 2-59 months in the family	1	346	84.4
	≥2	64	15.6
Number of family members	Two	6	1.5
	Three	106	25.8
	Four and above	298	72.7
Occupation of mothers/mothers	Housewife	87	21.2
	Merchant	108	26.3
	Student	44	10.7
	Government employee	54	13.2
	Non-governmental employee	24	5.9
	Farmers	87	21.2
	Others	6	1.5
Family monthly income	< 1000 ETB	244	59.5
	1001-2000 ETB	59	14.4
	2001 and above ETB	107	26.1
Age of child in months	≥12	212	51.7
	< 12	198	48.3
Gender of child	Male	232	56.6
	Female	178	43.4

### Health facility and illness related characteristics of the respondents

**Fig. 1:**
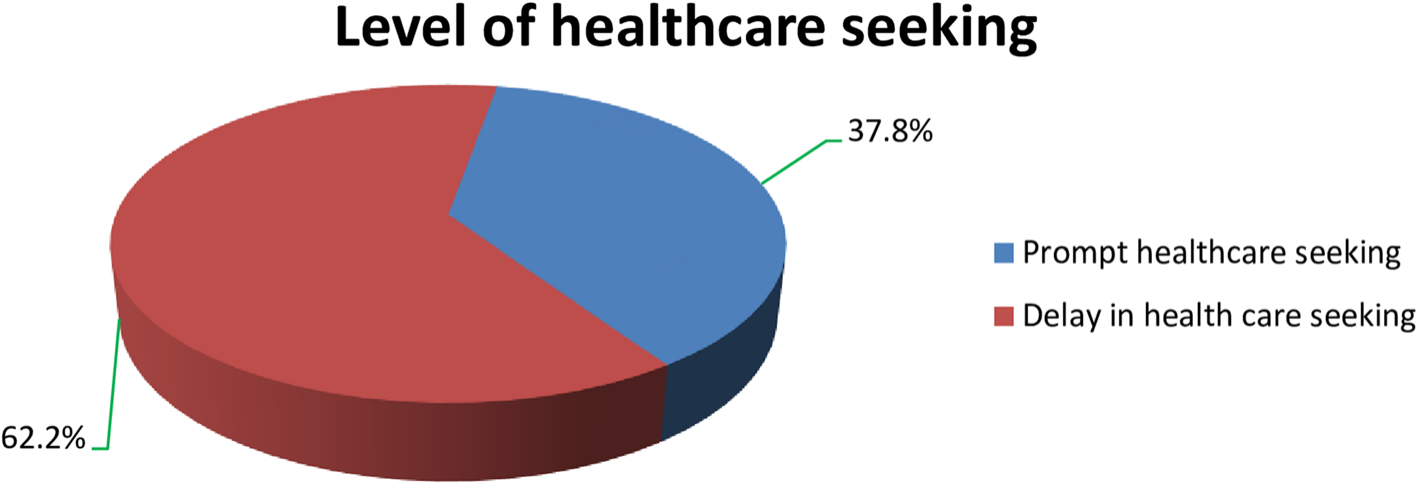
Level of healthcare seeking for pneumonia by mothers/caregivers of U5C

The prevalence of delay in seeking healthcare was 255 (62.2%) (Fig. 1). Among mothers or caregivers who preferred hospitals, 192 (46.73%) delayed seeking medical attention for their children who had pneumonia, and 115 (28.05%) sought medical attention within 24 hours of recognizing signs and symptoms. Mothers or caregivers who preferred public health centers and private clinics, 54 (13.17%) and 9 (2.2%), delayed visiting a health facility, respectively. More than half of the study participants took their children to the health facility on foot compared to those who came by vehicle, which is 246 (60%), of which 160 (39.02%) delayed coming early. of mothers/Caregivers who moved less than 1 h to reach the nearby health facility for healthcare seeking, 202 (49.27%) sought a health facility just after 24 hours of symptom recognition (Table.2).

**Table 2:** Health facility related characteristics of the study respondents in public health facilities in Nekemte town, Ethiopia, 2022

Variables	Delay in health care seeking	Prompt healthcare seeking	Total cases
**Type of health facility preferred for healthcare seeking**			
Public Hospital	192 (46.83%)	115 (28.05%)	307 (74.88%)
Public Health center	54 (13.17%)	32 (7.8%)	86 (20.98%)
Private clinic	9 (2.2%)	8 (1.96%)	17 (4.15%)
**The reason they preferred**			
Comprehensive exam	139 (33.9%)	92 (22.44%)	231 (56.34%)
Low waiting time	42 (10.24%)	19 (4.63%)	61 (14.88%)
Because it is nearby	20 (4.88%)	20 (4.88%)	40 (9.76%)
Drugs are well available	51 (12.44%)	23 (5.61%)	74 (18.05%)
open at any time	2 (0.49%)	0 (0%)	2 (0.49%)
Low cost to pay	1 (0.24%)	1 (0.24%)	2 (0.49%)
**Transportation type**			
On foot	160 (39.02%)	86 (20.98%)	246 (60%)
By vehicle	95 (23.17%)	69 (16.83%)	164 (40%)
**Distance of health facility from their residential area in minutes**			
< 60 minutes	202 (49.27%)	129 (31.46%)	331 (80.73%)
≥60 minutes	53 (12.93%)	26 (6.34%)	79 (19.27%)
**Distance of health facility from their residential area (in kilometers)**			
< 1	3 (0.73%)	10 (2.44%)	13 (3.17%)
1-1.9	7 (1.71%)	11 (2.68%)	18 (4.39%)
2-2.9	14 (3.41%)	27 (6.59%)	41 (10%)
3-3.9	20 (4.88%)	29 (7.07%)	49 (11.95%)
4-4.9	26 (6.34%)	44 (10.73%)	70 (17.1%)
≥5	85 (20.73%)	134 (32.68%)	219 (53.41%)
**Having community health insurance**			
Yes	38 (9.27%)	94 (22.93%)	132 (32.2%)
No	217 (52.9%)	61 (14.88%)	278 (67.8%)
**Have you treated this child at home?**			
Yes	76 (18.54%)	45 (10.98%)	121 (29.51%)
No	179 (43.66%)	110 26.83%)	289 (70.49%)
**If your answer to the question above is ‘yes’, how are you treated?**			
Using self-medication	72 (17.56%)	27 (6.59%)	99 (24.15%)
Using holly water	1 (024%)	6 (1.46%)	7 (1.7%)
Using traditional medicine	1 (0.24%)	7 (1.71%)	8 (1.95%)
Using special prayer	2 (0.49%)	5 (1.22%)	7 (1.7%)
**Decision makers for healthcare seek from the family**			
Father	66 (16.1%)	40 (9.76%)	106 (25.85%)
Mother	25 (6.1%)	9 (2.2%)	32 (8.3%)
Both partner	164 (40%)	106 (25.85%)	270 (65.85%)
**Signs and symptoms that lead the mother/caregiver to seek health care**			
Cough only	10 (2.44%)	6 (1.46%)	16 (3.9%)
Cough and fast breathing	106 (25.85%)	74 (18.05%)	180 (43.9%)
Cough and difficulty of breathing	15 (3.66%)	17 (4.15%)	32 (7.8%)
Cough and chest in drawing	14 (3.41%)	7 (1.71%)	21 (5.12%)
Cough and fever	110 (26.83%)	51 (12.44%)	161 (39.27%)
**How do you perceive the severity of illness?**			
Severe	40 (9.76%)	106 (25.85%)	146 (35.61%)
Moderate	12 (2.93%)	27 (6.59%)	39 (9.5%)
Mild	203 (49.5%)	22 (5.37%)	225 (54.88%)
**Does the child have an admission history previously**			
Yes	39 (9.5%)	14 (3.4%)	53 (12.93%)
No	116 (28.29%)	241 (58.78%)	357 (87.07%)
**What is the severity of pneumonia as diagnosed by physician?**			
Severe pneumonia	59 (14.39%)	34 (8.29%)	93 (22.68%)
Non-severe pneumonia	196 (47.8%)	121 (29.5%)	317 (77.32%)
**Is/are there a danger sign/signs (from card)?**			
Yes	57 (13.9%)	32 (7.8%)	89 (21.7%)
No	198 (48.3%)	123 (30%)	321 (78.3%)

### Mother’s/caregiver’s knowledge on pneumonia in children

Generally, around 386 (94.1%) mothers/caregivers have poor knowledge about pneumonia. Among study participants, only 182 (44.4%) know that pneumonia is a contagious disease, while 225 (54.9%) believe that pneumonia is a killer disease. Three-quarters (75.6%) of the respondents know that exclusive breastfeeding protects infants and younger children from pneumonia. Almost all 389 (94.9%) mothers/caregivers know that seeking healthcare within 24 hours of the onset of the signs and symptoms of pneumonia is better for saving a child’s life (Table 3).

**Table 3:** Respondents’ knowledge about pneumonia in children aged 2-59 months in public health facilities in Nekemte town, Ethiopia, 2022

Questions	Yes (%)	No (%)	I don’t know (%)
Pneumonia is a contagious disease.	182 (44.4)	154 (37.6)	74 (29)
Pneumonia is a killer disease.	225 (54.9)	105 (25.6)	80 (19.5)
Grunting can be a dangerous sign of pneumonia for children having cough.	194 (47.3)	96 (23.4)	120 (29.3)
A reduced level of consciousness can be a danger sign of pneumonia.	189 (46.1)	99 (24.1)	122 (29.8)
Chest in drawing is a dangerous sign of pneumonia.	154 (37.6)	125 (30.5)	131 (32)
Children get pneumonia easily if they don’t take immunization.	224 (54.6)	95 (23.2)	91 (22.2)
Undernourished child can easily get pneumonia.	268 (65.4)	72 (17.6)	70 (17.1)
Narrowed/non-ventilated/ house allows respiratory infection spread easily among family members.	263 (64.1)	83 (20.2)	64 (15.6)
Exclusive breastfeeding prevents children from getting pneumonia.	310 (75.6)	58 (14.1)	42 (10.2)
Cold/temperature/weather change can be a risk factor for pneumonia.	339 (82.7)	36 (8.8)	35 (8.5)
It is better to visit a health facility within 24 hours if the child is suspected of having pneumonia.	389 (94.9)	20 (4.9)	1 (0.24)
Do you have information on healthcare seeking for pneumonia?	65 (15.9)	345 (84.1)	0 (0)
Treating a child with pneumonia with unprescribed medicine is safe.	102 (24.9)	300 (73.2)	8 (2)
Did you treat pneumonia with home remedies?	72 (17.6)	338 (84.4)	0 (0)
Overall knowledge score	Poor	Good	
	94.1%	5.1%	

### Factors associated with delay in healthcare seeking for pneumonia in children aged 2-59 months

In multivariate logistic regression, rural residence, children aged ≥12 months, families’ low monthly income, caregivers not using health insurance, use of self-medication, poor knowledge, perceiving illness as mild, and having no previous admission history were significantly associated with delay in healthcare seeking among children aged 2–59 months with pneumonia.

The odds of delaying seeking healthcare for pneumonia are about three times higher among rural caregivers/mothers compared to urban residents (AOR = 2.77, 95% CI: 21.487–5.17). The finding also showed that the odds of a delay in healthcare for pneumonia are more than six times higher among families whose monthly income is less than 1000 ETB (AOR = 6.11, 95%CI: 2.16–17.26) compared to their counterparts. On the other hand, the odds of delay in seeking healthcare for pneumonia among children aged 12 months are 5.49 times (AOR = 5.49; 95% CI: 4.178–7.207) higher compared to < 12 months old.

Community health insurance is also a significantly associated factor for the delay, and the odds of delay among the non-users of health insurance is 8.93 times (AOR = 8.93; 95% CI: 5.43–14.68) more likely than the users. The odds of delay among the mothers and caregivers who use self-medication at home for their child’s illness are 10.97 times higher (AOR = 10.97; 95% CI: 1.85–65.3) compared to the use of special prayer before seeking healthcare (Table 4). Mothers who had poor knowledge of pneumonia in children were more delayed by 4.63 times (AOR = 4.63; 95% CI: 1.35–15.9) compared to those with good knowledge. In the same way, the odds of delay in seeking healthcare for pneumonia in children among mothers/caregivers who perceive the child’s illness as mild are 14.97 times (AOR = 14.97; 95% CI: 9.76–22.9) more likely than those who perceive the child’s disease as severe. Children with no hospitalization history are more likely than their counterparts to be delayed 2.85 times (AOR = 2.85; 95% CI: 1.77-4.56) (Table 4).

**Table 4:** Bivariate and multiple variable logistic regression analysis of factors associated with delay in healthcare seeking for pneumonia among children aged 2-59 months in public health facilities in Nekemte town, Ethiopia, 2022

Variables	Delay in seeking prompt healthcare for pneumonia		COR(95% CI)	AOR(95% CI)
	Yes	No		
**Residence**				
Urban	36 (8.78%)	59 (14.39%)	1.00	1.00
Rural	219 (53.41%)	96 (23.41%)	3.74 (2.32,6.04)*	2.77 (21.487,5.17)**
**Monthly income of the family in ETB**				
< 1000	177 (43.17%)	67 (16.34%)	3.64 (2.26,5.86)*	6.11 (2.16,17.26)**
1001-2000	33 (8.05%)	26 (6.34%)	1.75 (0.92,3.32)*	1.79 (0.455,7.022)
≥2001	45 (10.98%)	62 (15.12%)	1.00	1.00
**Age of child in months**				
≥12 months old	168 (40.95%)	44 (10.73%)	4.87 (3.15,7.52)	5.49 (4.178,7.207)**
< 12 months old	87 (21.22%)	111 (27.07%)	1.00	1.00
**Having community health insurance**				
Yes	94 (22.93%)	38 (9.27%)	1.00	1.00
No	61 (14.88%)	217 (52.9%)	8.8 (5.49,14.11)*	8.93 (5.436,14.68)**
**Home-based treatment child at home**				
Using self-medication	72 (17.56%)	27 (6.59%)	6.67 (1.22,36.44)*	10.97 (1.85,65.3)**
Using holly water	1 (024%)	6 (1.46%)	0.417 (0.03, 6.06)	0.71 (0.044,11.4)
Using traditional medicine	1 (0.24%)	7 (1.71%)	0.15 (0.03,0.94)	0.54 (0.04,8.26)
Using special prayer	2 (0.49%)	5 (1.22%)	1.00	1.00
**Knowledge of mothers/caregivers about pneumonia**				
Good knowledge	63 (15.37%)	27 (6.59%)	1.00	1.00
Poor knowledge	192 (46.8%)	128 (31.2%)	3.2 (1.077,9.58)*	4.63 (1.35,15.9)**
**Mothers’/caregivers’ perception on the severity of the illness**				
Severe	40 (9.76%)	106 (25.85%)	1.00	1.00
Moderate	12 (2.93%)	27 (6.59%)	1.178 (0.545,2.55)	1.53 (0.92,2.54)
Mild	203 (49.5%)	22 (5.37%)	24.5 (13.82,43.27*	14.97 (9.76,22.9)**
**Hospitalization history of the child**				
Yes	14 (3.4%)	39 (9.5%)	1.00	1.00
No	241 (58.78%)	116 (28.3%)	5.8 (3.02,11.08)*	2.85 (1.778,4.56)**

*AOR* Adjusted odds ratio, *COR* Crude odds ratio, *ETB* Ethiopian Birr; **: statistically significant at *p* < 0.05; *: statistically significant by COR at *p*-value< 0.25

## Discussion

This cross-sectional study aimed to determine the level of delay in seeking healthcare for pneumonia and associated factors among caregivers of U5C in public health facilities in Nekemte, Ethiopia. In this study, the proportion of delay in seeking healthcare for pneumonia in children aged 2–59 months is 62.2%. This finding is higher than the study conducted in Bangladesh (58%) [22] and Rwanda (56%) [23]. However, it is less than the study conducted in the Peruvian Amazon (65%) [24], Tanzania (87.2%) [25] and Kenya (66.1%) [26]. The difference might be due to variations in information access about healthcare, the availability and quality of healthcare services and healthcare workers, and the distance at which a healthcare facility is found from their home.

According to this study, early healthcare seeking for pneumonia in children is more commonly practiced by urban than rural mothers/caregivers. This finding is similar to Kenya [27] and Ethiopia, Bahir Dar [17]. This is likely because urban residents have better access to adequate information about pneumonia or health facilities in their local communities than rural residents. This demonstrates that rural residents delay taking an ill child with pneumonia to the hospital for one of two reasons: poor or insufficient information about pneumonia or a lack of a nearby facility. Providing adequate information to the rural community about pneumonia in U5C is valuable.

Mothers/caregivers with lower monthly incomes also initiated efforts to obtain prompt healthcare when their children showed features of pneumonia. This finding is supported by the study conducted in Kenya [26] and Ensaro District [20]. This might be due to the absence of money for treating the ill child. Besides, mothers/caregivers probably move to nearby towns to search for their daily income, which could expose them to a lack of transportation as there is a conflict due to political issues. This has an impact on their ability to seek treatment for pneumonia as soon as possible. Strengthening communities’ opportunity to use healthcare insurance is essential.

The study showed that there is a higher delay in seeking healthcare for pneumonia among younger children (12–59 months) than among infants (≤11 months). This is in harmony with the findings in Bangladesh [28], sub-Saharan Africa [29], and Tanzania [30]. From our experience, mothers/caregivers give more of their caring activities and time to the younger baby in the study area. Another reason could be that mothers and caregivers believe children over 1 year can tolerate illness better than their younger counterparts. Additionally, community health insurance prevents exposure to delays in early healthcare for pneumonia. This is in line with research findings from Bahir Dar, Ethiopia [17]. And it shows that if caregivers/mothers do not doubt treatment costs, they promptly visit healthcare facilities to treat their children for pneumonia. Encouraging caregivers/mothers to utilize community health insurance is suggested.

Moreover, self-medication is another reason for delays in seeking early healthcare for pneumonia. The result of this study was in line with a study conducted in Uganda [31]. The reason for this could be a lack of community awareness of healthcare seeking and a decrease in health information dissemination by health workers. In contrast, the study conducted in Rwanda [23] revealed that the significant predictors of delay in seeking care remained among parents’ special prayers. This might be due to the high perception of the community on their religion and poor awareness of the community in healthcare-seeking pneumonia in children aged 2-59 months.

Furthermore, mothers’ and caregivers’ lack of knowledge about pneumonia causes significant delays in prompt healthcare seeking. This is supported by research from Pakistan [32], Kenya [26], and Bahir Dar, Ethiopia [17]. This could be due to inadequate information or education about pneumonia. Improving mothers’ and caregivers’ knowledge of pneumonia is critical in U5C. Besides that, mothers/caregivers who believe pneumonia is a minor illness delay seeking medical attention earlier than those who do not think, so pneumonia is a minor illness. This research was similar to that done in the Philippines [33], Yemen [34], and Rwanda [23]. The information they had about pneumonia, whether from someone else or from their previous child’s illness (lesson learned), may have assisted the mothers/caregivers in visiting healthcare facilities. According to the findings of this study, mothers and caregivers with a history of hospitalization for their children visit healthcare facilities earlier. It is in line with study findings in Rwanda [23]. The reason might be due to the adequate information she acquired from the healthcare workers and other caregivers or the lessons learned from the negative consequences of pneumonia. It indicates that healthcare is a better place for getting correct information about pneumonia in children. Therefore, delivering the proper education or appropriate information about pneumonia to the mother/caregivers of the hospitalized child should be encouraged.

## Conclusion

In this study, the proportion of delay in seeking healthcare for pneumonia among children aged 2–59 months is high. Delay in seeking healthcare seeking was significantly and positively associated with rural residence, low monthly income, the age of the child (≥12 months), not using community health insurance, using self-medication at home, poor knowledge about pneumonia, a poor perception of the severity of the pneumonia illness, and previous admission status of a child. Creating caregivers’ awareness or providing adequate health education to develop early healthcare-seeking behavior at religious centers and through mass media and encouraging caregivers to use health insurance is essential.

### Limitations of the study

The study design is cross-sectional, which does not show a cause-and-effect relationship. The study excluded caregivers/mothers of children aged 2-59 months with pneumonia who did not visit health facilities. Recall bias is another limitation of the study, in which the caregivers were not responding to the exact time of disease onset in their children. On the other hand, this study mainly focused on public health facilities and in the tows, where community awareness is expected to be better than in rural areas and even where the availability of health facilities is greater. The associations observed among them could not identify whether the independent variable occurred before or after the occurrence of the dependent variable. Therefore, it is better to be conducted by other designs than cross-sectional to make it more clear association among them.

## Data Availability

All data generated or analyzed during this study are included in this published article and its supplementary information files.

## Abbreviations

AOR: Adjusted odds ratio
CHNRI: Child Health and Nutrition Research Initiative
COR: Crude odds ratio
DRC: Democratic Republic of Congo
ETB: Ethiopian Birr
OPD: Outpatient department
SPSS: Statistical Package for Social Scientist
UNICEF: United Nations International Children emergency Fund
U5C: Under Five Children
WHO: World Health Organization
